# The incidence and risk factors of meconium amniotic fluid in singleton pregnancies: an experience of a tertiary hospital in Iran

**DOI:** 10.1186/s12884-022-05285-8

**Published:** 2022-12-12

**Authors:** Mitra Shekari, Malihe Shirzadfard Jahromi, Amene Ranjbar, Vahid Mehrnoush, Fatemeh Darsareh, Nasibeh Roozbeh

**Affiliations:** 1 Mother and Child Welfare Research Center, Hormozgan University of Medical Sciences, Bandar Abbas, Iran; 2 Fertility and Infertility Research Center, Hormozgan University of Medical Sciences, Bandar Abbas, Iran

**Keywords:** Meconium, Amniotic fluid, Risk factors

## Abstract

**Background:**

Several common maternal or neonatal risk factors have been linked to meconium amniotic fluid (MAF) development; however, the results are contradictory, depending on the study. This study aimed to assess the prevalence and risk factors of MAF in singleton pregnancies.

**Methods:**

This study is a retrospective cohort that assessed singleton pregnant mothers who gave birth at a tertiary hospital in Bandar Abbas, Iran, between January 1st, 2020, and January 1st, 2022. Mothers were divided into two groups: 1) those diagnosed with meconium amniotic fluid (MAF) and 2) those diagnosed with clear amniotic fluid. Mothers with bloody amniotic fluid were excluded. Demographic factors, obstetrical factors, and maternal comorbidities were extracted from the electronic data of each mother. The Chi-square test was used to compare differences between the groups for categorical variables. Logistic regression models were used to assess meconium amniotic fluid risk factors.

**Results:**

Of 8888 singleton deliveries during the study period, 1085 (12.2%) were MAF. MAF was more common in adolescents, mothers with postterm pregnancy, and primiparous mothers, and it was less common in mothers with GDM and overt diabetes. The odds of having MAF in adolescents were three times higher than those in mothers 20–34 years old (aOR: 3.07, 95% CI: 1.87–4.98). Likewise, there were significantly increased odds of MAF in mothers with late-term pregnancy (aOR: 5.12, 95% CI: 2.76–8.94), and mothers with post-term pregnancy (aOR: 7.09, 95% CI: 3.92–9.80). Primiparous women were also more likely than multiparous mothers to have MAF (aOR: 3.41, 95% CI: 2.11–4.99).

**Conclusions:**

Adolescents, primiparous mothers, and mothers with post-term pregnancies had a higher risk of MAF. Maternal comorbidities resulting in early termination of pregnancy can reduce the incidence of MAF.

## Background

Meconium can be found in fetuses' gastrointestinal tracts as early as 14–16 weeks gestation [1]. Meconium amniotic fluid (MAF) occurs when there is a passage of the fetal colonic contents into the amniotic cavity [2]. Although water makes up 85–95% of meconium, the remaining 5–15% comprises gastric secretions, bile salts, mucus, vernix, lanugo, blood, pancreatic enzymes, free fatty acids, and squamous cells [3]. Intrauterine meconium passage in near-term or term fetuses has been linked to fetomaternal stress factors and/or infection, whereas post-term meconium passage has been linked to gastrointestinal maturation [1]. MAF is common among women in spontaneous labor at term, with a prevalence of 15% of pregnancies [4]. MAF causes are thought to be relatively complex; additionally, the pathophysiology of this condition has not yet been completely elucidated [4]. Several studies have linked MAF to an obstetric risk and a significant increase in the risk of adverse neonatal outcomes [5, 6]. Intrauterine meconium exposure is associated with inflammation of the lung, chorionic plate, and umbilical vessel tissues, which may contribute to neonatal morbidity through various mechanisms [7]. These findings highlight the importance of identifying MFA risk factors. Most MAF studies focus on prenatal outcomes, and little is known about the risk factors that put pregnant mothers at increased risk of developing MAF. This study aimed to assess the prevalence and risk factors of MAF in singleton pregnancies.

## Methods

### Study design

This study is a retrospective cohort that assessed singleton pregnant mothers who gave birth at Khaleej-e-Fars Hospital (a tertiary hospital) in Bandar Abbas, Iran, between January 1st, 2020, and January 1st, 2022. This study complies with the Declaration of Helsinki and was performed according to ethics committee approval. Statistical analysis was performed with patient anonymity.

### Participants

Mothers were divided into two groups: 1) those diagnosed with MAF and 2) those diagnosed with clear amniotic fluid (CAF). Mothers with bloody amniotic fluid were excluded.

### Data collection

Using electronic patient records, data were extracted by trained collectors from the "Iranian Maternal and Neonatal Network (IMaN Net)," a valid national system. Demographic factors (age, educational level, place of residency, medical insurance, access to prenatal care facilities, smoking status), obstetrical factors (gestational age, parity, newborn sex, oligohydramnios, preeclampsia, gestational diabetes mellitus (GDM), abnormal placentation, placental abruption, chorioamnionitis, intrauterine growth restriction (IUGR), intrauterine fetal death (IUFD)), and maternal comorbidities (overt diabetes mellitus, chronic hypertension, cardiovascular disease, thyroid dysfunction, drug addiction, hepatitis, anemia [8], infertility, and COVID-19 at the time of admission) were extracted from the electronic data of each mother.

### Data management and analysis

The IBM Statistical Package for the Social Sciences Statistics, version 25, was used to examine the data (IBM Corp, Armonk, NY). Categorical variables are presented as numbers and frequencies (%). The Chi-square test was used to compare differences between the groups for categorical variables. To identify the associated factors of MAF, multiple logistic regressions were fitted for MAF, and odds ratios (OR) with 95% confidence intervals (95% CI) were calculated. Variables with *p* values less than 0.2 were kept in the model as potential confounders for multivariable analysis. The Hosmer‒Lemeshow goodness-of-fit test with a 95% confidence interval was used for multivariable analysis. Following analysis, a backward conditional selection method was used until all of the remaining variables were determined to be significant with a *p* value of 0.05.

## Results

Of 8888 singleton deliveries during the study period, 1085 (12.2%) were MAF. The association between demographic, obstetrical, and medical history and MAF was assessed. Factors that showed an association in the chi-square analysis were age (Table 1), gestational age, parity, GDM, and overt diabetes (Table 2). MAF was more common in adolescent mothers, mothers with a gestational age of more than 40 weeks, and primiparous mothers, and it was less common in mothers diagnosed with GDM and overt diabetes.

**Table 1:** Maternal characteristics of women diagnosed with meconium amniotic fluid

Demographic characteristics	CAF (*n* = 7803)	MAF (*n* = 1085)	Total (*n* = 8888)	*P* value
**Age (Years)**				< 0.001
13–19	441 (5.7)	93 (8.6)	534 (6)	
20–34	5688 (72.9)	810 (74.7)	6498 (73.1)	
35 and above	1674 (21.5)	181 (16.7)	1855 (20.9)	
**Educational level**				0.058
Illiterate	481 (6.2)	76 (7)	557 (6.3)	
Elementary	2398 (30.7)	332 (30.6)	2730 (30.7)	
High school/Diploma	3624 (46.4)	467 (43)	4091 (46)	
Advanced	1300 (16.7)	210 (19.4)	1510 (17)	
**Residency place**				0.999
Urban	5179 (66.4)	720 (66.4)	5899 (66.4)	
Rural	2624 (33.6)	365 (33.6)	2989 (33.6)	
**Access to prenatal care**				
Yes	7738 (99.2)	1073 (98.9)	8811 (99.1)	
No	65 (0.8)	12 (1.1)	77 (0.9)	0.380
**Medical insurance**				0.561
Yes	7582 (97.2)	1051 (96.9)	8633 (97.1)	
No	221 (2.8)	34 (3.1)	255 (2.9)	
**Smoking**				0.761
Yes	17 (0.2)	4 (0.4)	21 (0.2)	
No	7786 (99.8)	1081 (99.6)	8867 (99.8)	

Data are presented as n (%)

*MAF* Meconium amniotic fluid, *CAF* Clear amniotic fluid

**Table 2:** Obstetrical and medical characteristics of mothers diagnosed with meconium amniotic fluid

Variables		CAF (*n* = 7803)	MAF (*n* = 1085)	Total (*n* = 8888)	*P* value
**Obstetrical**	**Gestational age**				< 0.001
	Less than 37 weeks	1190 (15.3)	55 (5.1)	1245 (14)	
	37–40 weeks	5685 (72.9)	751 (69.2)	6436 (72.4)	
	40^+1^–41 weeks	779 (10)	236 (21.8)	1015 (11.4)	
	More than 41 weeks	149 (1.9)	43 (4)	192 (2.2)	
	**Parity**				< 0.001
	Primiparous	2026 (26)	481 (44.3)	2507 (28.2)	
	Multiparous (2–5 parity)	5572 (71.4)	572 (52.7)	6144 (69.1)	
	Grand multiparous (6 parity and more)	205 (2.6)	32 (2.9)	237 (2.7)	
	**Oligohydramenios**				0.091
	No	7490 (96)	1016 (93.6)	8506 (95.9)	
	Yes	313 (4)	69 (6.4)	382 (3.7)	
	**Newborn Sex**				0.088
	Female	3827 (49)	503 (46.4)	4330 (48.7)	
	Male	3976 (51)	582 (53.6)	4858 (51.3)	
	**Preeclampsia**				0.599
	No	7292 (93.5)	1019 (9.9)	8311 (93.5)	
	Yes	511 (6.5)	66 (6.1)	577 (6.5)	
	**Placenta abruption**				0.927
	No	7553 (96.8)	1050 (96.8)	8603 (96.8)	
	Yes	250 (3.2)	35 (3.2)	285 (3.2)	
	**Placenta abnormalities**				0.309
	No	7769 (99.6)	1083 (99.8)	8852 (99.6)	
	Yes	34 (0.4)	2 (0.2)	36 (0.4)	
	**Chorioamnionitis**				0.423
	No	7772 (99.6)	1083 (99.8)	8855 (99.6)	
	Yes	31 (0.4)	2 (0.2)	33 (0.4)	
	**Intrauterine growth retardation**				0.789
	No	7450 (96.6)	1059 (97.6)	8599 (96.7)	
	Yes	263 (3.4)	26 (2.4)	289 (3.3)	
	**Intrauterine fetal death**				0.999
	No	7769 (99.6)	1081 (99.6)	8850 (99.6)	
	Yes	34 (0.4)	4 (0.4)	38 (0.4)	
	**Gestational diabetes**				< 0.001
	No	6620 (84.4)	950 (87.6)	7570 (85.2)	
	Yes	1183 (15.6)	135 (12.4)	1318 (14.8)	
**Comorbidities**	**Infertility**				0.379
	No	7780 (99.7)	1080 (99.5)	8860 (99.7)	
	Yes	23 (0.3)	5 (0.5)	28 (0.3)	
	**Anemia**				0.454
	No	7569 (97)	1062 (97.9)	8631 (97.1)	
	Hemoglobin 7–10	133 (1.7)	13 (1.2)	146 (1.6)	
	Hemoglobin less than 7	101 (1.3)	10 (0.9)	111 (1.3)	
	**Cardiovascular disease**				0.343
	No	7716 (98.9)	1077 (99.3)	8793 (98.9)	
	Yes	87 (1.1)	8 (0.7)	95 (1.1)	
	**Pyelonephritis**				0.607
	No	7795 (99.9)	1085 (100)	8880 (99.9)	
	Yes	8 (0.1)	0	8 (0.1)	
	**Drug addiction**				0.201
	No	7755 (99.4)	1083 (99.8)	8838 (99.5)	
	Yes	48 (0.6)	2 (0.2)	50 (0.5)	
	**Chronic Hypertension**				0.063
	No	7711 (98.8)	1079 (99.4)	8790 (98.9)	
	Yes	92 (1.2)	6 (0.6)	98 (1.1)	
	**COVID-19**				0.189
	No	7687 (98.5)	1053 (98)	8750 (98.4)	
	Yes	116 (1.5)	22 (2)	138 (0.6)	
	**Overt Diabetes**				0.049
	No	7778 (99.6)	1085 (100)	8858	
	Yes	30 (0.4)	0	30	
	**Thyroid dysfunction**				0.751
	No	6985 (89.5)	968 (89.2)	7953 (89.5)	
	Yes	818 (10.5)	117 (10.8)	935 (10.5)	
	**Hepatitis**				0.986
	No	7775 (99.6)	1082 (99.7)	8857 (99.7)	
	Yes	28 (0.4)	3 (0.3)	31 (0.3)	

Data are presented as numbers (%)

*MAF* Meconium amniotic fluid, *CAF* Clear amniotic fluid

Table 3 represents the risk factors of MAF based on logistic regression analysis. In this study, the association between demographic, obstetrical, and maternal morbidities and MAF was assessed. The variables that showed an association in the bivariable analysis were age, gestational age, parity, and gestational and overt diabetes. These variables were used for multivariable analysis to adjust for confounding factors. The adjusted odds ratio (AOR) revealed that age, gestational age, and parity were significantly associated with MAF. The odds of having MAF in adolescents were three times higher than those in mothers 20–34 years old (aOR: 3.07, 95% CI: 1.87–4.98). Likewise, there were significantly increased odds of MAF in mothers with late-term pregnancy (aOR: 5.12, 95% CI: 2.76–8.94), and mothers with post-term pregnancy (aOR: 7.09, 95% CI: 3.92–9.80). Primiparous women were also more likely than multiparous mothers to have MAF (aOR: 3.41, 95% CI: 2.11–4.99).

**Table 3:** Factors associated with meconium amniotic fluid

Variables	COR (95% CI)	AOR (95% CI)
**Age**		
13–19	4.25 (2.13–7.01)*	3.07 (1.87–4.98)*
20–34	1	1
35 and more	0.86 (0.43–1.01)	0.92 (0.38–1.23)
**Gestational age**		
Less than 37 weeks	0.45 (0.12–0.93)*	0.57 (0.25–0.77)*
37–40 weeks	1	1
40^+1^–41 weeks	5.89 (2.3–9.8)*	5.12 (2.76–8.94)*
More than 41 weeks	7.12 (3.04–9.97)*	7.09 (3.92–9.8)*
**Parity**		
1	3.46 (1.01–5.16)*	3.41 (2.11–4.99)*
2–5	1	1
6 and more	1.68 (0.84–2.11)	1.75 (1.4–1.98)
**Gestational diabetes**		
Yes	0.44 (0.23–0.87)*	0.79 (0.33–0.91)
No	1	1
**Overt diabetes**		
Yes	0.42 (0.24–0.74)*	0.71 (0.35–0.98)
No	1	1

^*^P < 0.05

## Discussion

The relationship between demographic, obstetrical, and maternal morbidities and MAF was investigated in this study. Age, gestational age, parity, and gestational and overt diabetes were the variables that showed an association in the bivariable analysis. Multivariable analysis revealed that MAF was significantly associated with age, gestational age, and parity. MAF has been reported to affect 24.6% of Ethiopians [9], 8.3% of Indians [10], and 20.4% of Nigerians [11]. We were unable to locate any records about the prevalence of MAF in Iran. The incidence of MAF in our study was 12.2%, slightly lower than what has been reported globally [4]. The fact that most women in our study (97.8%) had a gestational age of 41 weeks or less may explain the low incidence of MAF. Our study found that maternal age was a significant risk factor for MAF. Adolescents were three times more likely than mothers aged 20 to 34 to develop MAF, which is a new finding. Previously published studies identified older maternal age as an independent risk factor for MAF [8, 9]. In contrast, most studies found no link between maternal age and MAF [6, 9, 12]. This could be due to study design, setup, and population differences. More research is required to explain the controversy.

Gestational age was the other independent risk factor for MAF. It has been shown that MAF increases steadily with increasing gestational age. MAF has been found in 5% of pregnancies before 37 weeks, 25% of term pregnancy births, and up to 52% of post-term pregnancies [4]. Several population-based cohort studies showed that gestational age at delivery was independently associated with MAF [6, 8, 9]. This might be explained by the maturation of the gastrointestinal tract and increased secretion of motilin by the fetus as gestational age advances, leading to increased fetal bowel peristalsis ending up in the passage of meconium [4].

Parity was a significant predictor of MAF, with primiparous mothers being three times more likely to develop MAF. Having childbirth previously has been shown to be a protective factor against MAF occurrence [6]. In comparison, some studies found no association between parity and MAF [9, 13]. According to Patel et al., grade 1 MAF (thin meconium) fluid is more common in multigravida mothers, whereas grade 2 and 3 MAF (thick meconium) are more common in primigravida patients [14]. Further investigation is required to obtain more in-depth information regarding this issue.

Regarding obstetrical factors, several studies have identified IUGR and oligohydramnios as significant risk factors for MAF [15, 16]; however, our findings contradict previous literature. Other obstetrical factors were also not associated with MAF, including placental abnormalities, placental abruption, IUFD, and chorioamnionitis.

In terms of maternal comorbidities, various studies have linked various maternal comorbidities to an increased risk of MAF. For example, Gupta et al. discovered a higher incidence of MAF in mothers with hepatitis [15]. Pregnancy-induced hypertension and preeclampsia have also been identified as MAF risk factors [9]. The link between hypertension and meconium passage has been linked to uteroplacental insufficiency, which causes fetal hypoxia and meconium passage [17]. Our findings, however, found no link between MAF and maternal comorbidities (chronic hypertension, preeclampsia, pyelonephritis, anemia, infertility, COVID-19, hepatitis, drug addiction, and cardiovascular disease). This discrepancy is because most maternal comorbidities indicate the need to terminate a pregnancy at an early gestational age. As a result, few mothers with comorbidities have a post-term pregnancy.

The only comorbidities linked to MAF were overt diabetes and gestational diabetes, with a lower incidence of MAF in mothers with a history of overt or gestational diabetes. Most diabetes guidelines recommend elective birth via labor induction around the estimated delivery date and earlier (at 38–39 weeks of pregnancy) if there are any maternal or fetal complications [18]. As a result, the incidence of post-term pregnancy, a significant risk factor for MAF, decreases in overt and gestational diabetic mothers. It should be mentioned that after adjusting for confounders, no link was observed between diabetes and MAF.

Investigation of the prevention factors of meconium aspiration is frequently mentioned in the literature review. However, few studies have been conducted in the field of factors influencing meconium occurrence prevention. The main reason for this is that meconium cannot be detected before labor begins. On the other hand, many variables related to the occurrence of meconium, such as those examined in this study, are uncontrollable in most cases, such as age and number of births. Post-term pregnancy is the most important known factor in the occurrence of MAF, so reducing the number of cases of post-term pregnancy can be an important determining factor in the prevention of MAF. It should be noted that, while international guidelines recommend terminating a pregnancy at 42 weeks if there are no special problems [2], Iranian guidelines recommend terminating a pregnancy at 41 weeks, especially if the cervix is suitable for delivery [19]. This has the potential to reduce many cases of MAF. More clinical trials are needed to determine the effect of early pregnancy termination on the incidence of MAF.

The strength of our study is that our study registers are of high quality and in accordance with childbirth records. We investigated various factors associated with MAF in pregnancies. The population study sample size was large enough to reflect the situation regarding identifying risk factors of MAF. Our study was conducted retrospectively, which is still a limitation. The database did not allow for the precise timing of the various events during pregnancy. Another limitation of our study is that we did not perform a subanalysis of MAF severity. More data were missing for variables such as body mass index and the length of labor. Because we did not have access to data on the durations of labor and cardiotocography of all study participants, we were unable to assess the risk of MAF in relation to the duration of labor or abnormal cardiotocography. This should be considered in future studies, especially since the most recent study found that the duration of labor and pathologic cardiotocography affect the risk of fetal acidemia [20]. Excluding mothers with bloody amniotic fluid from the study may result in selection bias. A proportion of mothers with bloody amniotic fluid could be MAF at the same time. It was preferable to include those mothers in the study and conduct a subanalysis. Future research should take this into account.

## Conclusions

MAF is associated with maternal age, parity, and gestational age. Adolescents, primiparous mothers, and mothers with post-term pregnancies had a higher risk of MAF. Maternal comorbidities resulting in early termination of pregnancy can reduce the incidence of MAF. There are currently no MAF prevention strategies to be implemented. Identifying the risk factors that put pregnant mothers at a higher risk of developing MAF, on the other hand, may help us focus more attention on the high-risk group to prevent the negative outcomes of MAF. We believe that the current study's findings will aid professionals in developing preventive strategies.

## Data Availability

The datasets generated and/or analyzed during the current study are available from the corresponding author upon reasonable request.

## Abbreviations

MAF: Meconium amniotic fluid
CAF: Clear amniotic fluid
LBW: Low birth weight
IUFD: Intrauterine fetal death
IUGR: Intrauterine growth retardation
GDM: Gestational diabetes mellitus
